# P-910. Enhancing Awareness: Early Detection of Varicella-Zoster in CNS Among Immunocompromised Patients

**DOI:** 10.1093/ofid/ofae631.1101

**Published:** 2025-01-29

**Authors:** Estefania Sienra-Iracheta, Bianca Paola Aguilar Rodea, Angela Michelle Arias Lopez, Melissa Mariana Lopez Ramos, Juan Pablo Ramírez-Hinojosa, Juan Pablo Venzor Castellanos, Patricia Rodriguez-Zulueta, Xochitl Yadira Sanchez Godinez, David Medina Julio

**Affiliations:** Hospital General Dr. Manuel Gea González, Mexico City, Distrito Federal, Mexico; Hospital General Dr. Manuel Gea Gonzalez, CDMX, Distrito Federal, Mexico; Hospital General Dr. Manuel Gea Gonzalez, CDMX, Distrito Federal, Mexico; Hospital General Dr. Manuel Gea Gonzalez, CDMX, Distrito Federal, Mexico; Hospital General Dr. Manuel Gea Gonzalez, CDMX, Distrito Federal, Mexico; Hospital General Dr. Manuel Gea Gonzalez, CDMX, Distrito Federal, Mexico; Hospital General Dr. Manuel Gea González, Mexico City, Distrito Federal, Mexico; Hospital General Dr. Manuel Gea Gonzalez, CDMX, Distrito Federal, Mexico; Hospital General Dr. Manuel Gea Gonzalez, CDMX, Distrito Federal, Mexico

## Abstract

**Background:**

Varicella zoster virus (VZV) reactivation typically presents as herpes zoster, but central nervous system (CNS) involvement is more prevalent in immunocompromised individuals.

**Methods:**

This retrospective study, conducted from January 2023 to March 2024 at two hospitals, aimed to characterize VZV CNS infections in this population. Twenty-seven immunocompromised patients with active herpes zoster were included. Clinical data, cerebrospinal fluid (CSF) analysis, and treatment outcomes were analyzed.

**Results:**

The mean age was 45 years, with a predominance of males (60%). Most patients had HIV (16) with a mean CD4 count of 126, while others had cancer (5), autoimmune diseases (2), or diabetes mellitus (1). Headache was the most common symptom (64%), followed by fever (19%), altered consciousness, and behavioural changes (7%). Facial palsy was observed in 3 patients, but no seizures were reported. CSF analysis revealed hyperproteinorrachia (mean 75 mg/dL) and pleocytosis (mean 39 cells). All patients received intravenous acyclovir for an average of 14 days, with a 100% survival rate at 30 days.

**Conclusion:**

This study highlights the importance of early suspicion and diagnosis of VZV CNS infections in immunocompromised individuals, even in the absence of classical neurological symptoms. Prompt initiation of intravenous acyclovir therapy is crucial for favourable outcomes.

**Disclosures:**

**Estefania Sienra-Iracheta, MD**, PFIZER: Advisor/Consultant|STENDHAL: Advisor/Consultant **Juan Pablo Ramírez-Hinojosa, MD**, Abott: Honoraria|Jansen: Honoraria|Silanes: Honoraria **Patricia Rodriguez-Zulueta, MD**, PFIZER: Advisor/Consultant|STENDHAL: Advisor/Consultant

